# Reliability of self-reported toothbrushing frequency as an indicator for the assessment of oral hygiene in epidemiological research on caries in adolescents: a cross-sectional study

**DOI:** 10.1186/s12874-015-0002-5

**Published:** 2015-03-08

**Authors:** Giovana S Gil, Francine S Morikava, Gabriela C Santin, Tatiana P Pintarelli, Fabian C Fraiz, Fernanda M Ferreira

**Affiliations:** Department of Stomatology, Federal University of Parana, Av. Prefeito Lothário Meissner, 632 Curitiba, Brazil

**Keywords:** Toothbrushing, Adolescent, Dental plaque indices, Self report

## Abstract

**Background:**

In the present state of knowledge regarding the etiology of dental caries, it is unacceptable for studies addressing factors associated with this outcome to disregard oral hygiene. Simple, valid methods are needed for the assessment of oral hygiene in adolescents to allow this condition to be properly investigated in epidemiological studies on caries and assist in the establishment of health promotion measures. The aim of the present study was to test the hypothesis that the self-reported toothbrushing frequency can be used as a proxy measure for clinical oral hygiene indices in epidemiological studies on dental caries in adolescents.

**Methods:**

A cross-sectional study was conducted with a representative sample (n = 589) of 12-year-old school children in a medium-sized city in southern Brazil. A detailed questionnaire addressing socio-demographic and economic characteristics was sent to primary caregivers. Adolescents answered a brief self-administered questionnaire on behavioral characteristics, including toothbrushing frequency and sugar intake. Samples of stimulated saliva were collected from the adolescents and evaluated for levels of mutans streptococci and lactobacilli using Dentacult kits I and II, respectively. Examiners who had undergone a training and calibration exercise (kappa > 0.81) performed the clinical examination of the adolescents. Caries experience was assessed using the decayed, missing and filled teeth index. Oral hygiene was determined using the Simplified Oral Hygiene Index and the Visible Plaque Index.

**Results:**

When the oral hygiene variables were used alone in the multiple models, significant associations with dental caries were found. When Simplified Oral Hygiene Index and/or Plaque Index were used together with toothbrushing frequency in the same model, only the latter was significantly associated with dental caries. A significant association was also found between self-reported toothbrushing frequency and the clinical indices.

**Conclusion:**

Based on the present findings, self-reported toothbrushing frequency can be used as a proxy measure for clinical oral hygiene indices, which facilitates data collection in epidemiological studies addressing dental caries in adolescents.

## Background

Although preventable, dental caries is considered the most common adverse oral condition in childhood and adolescence [1]. In early adolescence, young people exhibit behavior and face circumstances that make them more vulnerable to dental caries, such as a low frequency of daily brushing [2] and the excessive consumption of cariogenic foods and beverages [3]. Thus, adolescence is an important period for interventions aimed at the establishment of healthy habits for promoting general and oral health [4].

Oral hygiene habits have been associated with dental caries experience in 12-year-old adolescents [2,5]. A higher toothbrushing frequency seems to reduce the risk of this condition [6-8]. Therefore, in the present state of knowledge regarding the etiology of dental caries, it is unacceptable for studies addressing factors associated with this outcome to disregard oral hygiene.

Simple, valid methods are needed for the assessment of oral hygiene in adolescents to allow this condition to be properly investigated in epidemiological studies. Clinical indicators are considered more reliable measures in health studies. Oral hygiene is commonly assessed through clinical indices, such as the Simplified Oral Health Index (OHI-S) [9] and the Visible Plaque Index (VPI) [10]. However, these assessment tools require a detailed clinical examination. Simplified methods, such as self-reported toothbrushing frequency, have also been used to measure oral hygiene.

The reliability of self-reported measures in epidemiological studies depends on many factors, such as the age of the interviewee. David et al. [11] found that self-reports from 12-year-old adolescents are reliable in the assessment of oral health. However, no previous studies are found in the literature addressing the possibility of using self-reported toothbrushing frequency by adolescents as an indicator of oral hygiene in place of clinical measures. Thus, the aim of the present study was to test the hypothesis that self-reported toothbrushing frequency can be used as a proxy measure for clinical oral hygiene indices in epidemiological studies on dental caries in adolescents.

## Methods

### Study design and sample

A cross-sectional study was conducted with a representative sample of male and female 12-year-old schoolchildren in a medium-sized city in southern Brazil. Two-stage (schools and adolescents), randomized, cluster sampling was performed. To ensure representativeness, the sample was selected from public and private schools in the eight administrative districts of the city, maintaining the proportion of 12-year-old students enrolled in each district. One public and one private school were randomly selected from each district to obtain the required proportional number of students. After the definition of the schools, students were randomly selected. The sample size was calculated based on the formula for estimation of proportion, using a 63.3% prevalence rate of dental caries among 12-year-old schoolchildren in southern Brazil [12], a 95% confidence level and a 5% tolerable error. The sample was then increased by 50% for the design effect and an additional 20% to compensate for possible losses, totalling 644 12-year-old students.

Schoolchildren wearing a fixed orthodontic appliance, those with any signs illness at the time of data collection (fever above 100.4°F, diarrhea, runny nose, vomiting) and those absent from school on the days scheduled for the examinations were excluded from the study.

### Data collection

#### Socio-demographic and economic data

A detailed questionnaire addressing socio-demographic and economic characteristics was sent to the primary caregivers. Mother’s schooling and household income (measured in terms of the Brazilian monthly minimum salary, which corresponded to about US$ 290.47 at the time of the study) were assessed.

#### Behavioral data

The adolescents answered a brief self-administered questionnaire on behavioral characteristics, including toothbrushing frequency and daily frequency of sugar intake. Daily toothbrushing frequency was assessed using a single item: “At what times of the day do you brush your teeth?”. The response options were subsequently dichotomized for the statistical analysis into “more than two brushings per day” or “up to two brushings per day”, as proposed by Jurgensen and Petersen [13]. For the assessment of dietary habits, a specific food frequency questionnaire was used. This questionnaire was developed in a pilot study for the quantification of the number of sugary foods and beverages consumed daily.

#### Salivary levels of mutans streptococci and lactobacilli

Samples of stimulated saliva were collected from the adolescents and evaluated for levels of mutans streptococci and lactobacilli using Dentacult kits I and II, respectively (Laborclin™, Pinhais, PR, Brazil) [14]. The volunteers were instructed to chew a paraffin substance measuring 2 cm^2^ (Parafilm M™, Laboratory Film, Chicago, USA), swallowing the saliva produced normally in the first minute and spitting the saliva produced in the second and third minutes into a disposable plastic container.

An aliquot of saliva was immediately used for the inoculation of the culture medium on the surfaces of the slides of Dentalcult kits I and II. A disc of bacitracin was then placed on the surface of the culture medium of Dentalcult II and a CO2 tablet was placed at the bottom of the recipient. The vials were sealed and transported to the laboratory, placed in an incubator for microbiological cultures at 98.6°F for 48 hours (Dentalcult II) or 72 hours (Dentalcult I). At the end of the incubation period, the number of colony forming units of mutans streptococci/mL and lactobacilli/mL was calculated for each sample by the similarity with bacterial levels illustrated on the template provided by the manufacturer.

#### Clinical data

The examiners underwent training and calibration exercises for the collection of the clinical data (diagnosis of dental caries and determination of plaque indices). The calibration exercise consisted of two steps. The first was a theoretical step procedure that involved discussion of the criteria for the diagnosis and analysis of photographs of the conditions. A dentist with experience in epidemiological surveys (gold standard in this theoretical framework) coordinated this step, training one general dentist (TPP) in how to perform the examination. The second step was the clinical part, at which the dentists examined 12 previously selected 12-year-old adolescents. Inter-examiner agreement was tested comparing the examiner with the gold standard. After 15 days, the examiner repeated the exam for dental caries. For plaque indices, it was only possible to determine the inter-examiner agreement. Data analysis used the Cohen’s kappa coefficient on a tooth-by-tooth basis. Very good intra e inter-examiner agreements were obtained and all values of Kappa were greater than 0.81, which is satisfactory for this type of assessment [15].

The clinical examinations were performed at each school by a single examiner, who was blinded to previous collected data at that moment, under natural light with the adolescent sitting in school chair, leaning his/her head back, following the international criteria standardized by the World Health Organization for research in oral health [15]. The examining dentists used personal protective equipment and sterilized, packaged clinical kits containing a dental mirror, periodontal probe (WHO-621) and gauze.

Oral hygiene was assessed using the “plaque” component of the Simplified Oral Health Index (OHI-S), according to the diagnostic criteria of the World Health Organization [15] and was dichotomized as “satisfactory” (score: <1) or “unsatisfactory” (score: ≥ 1). The Visible Plaque Index (VPI) was used to determine the frequency of tooth surfaces covered with clearly visible plaque [10], the outcome of which was dichotomized as “present” (at least one surface covered with visible dental plaque on the buccal region of the maxillary anterior teeth) or “absent” (dental plaque not visible on any surface of the buccal region of the maxillary anterior teeth). Caries experience was assessed using the decayed, missing and filled teeth (DMFT) index, which is the traditional caries index for the permanent dentition [15].

### Statistical analysis

Bivariate analysis (Mann–Whitney and Kruskal-Wallis tests) was first performed for dental caries (DMFT scores) and the explanatory variables. The data were then analyzed using multiple Poisson regression, with robust variance. This strategy allowed estimating rate ratios (ratio of arithmetic means) and respective 95% confidence intervals of DMFT scores for comparisons between self-reported toothbrushing frequency and the clinical oral hygiene indices, controlled for other covariates. The explanatory variables were dichotomized based on theoretical references or by the median. Variables with a p-value < 0.20 in the bivariate analysis were incorporated into the multivariate models and those with p-value < 0.05 remained in the final model. In each of the first three models, oral hygiene was assessed by one variable (Model 1: self-reported toothbrushing frequency; Model 2: Simplified Oral Hygiene Index; Model 3: Visible Plaque Index). Three other models were run to estimate the strength of associations between the DMFT index and self-reported toothbrushing frequency or clinical oral hygiene indices, with the oral hygiene variables incorporated in pairs (Model 4 and 5) as well as all three measures incorporated in the same model (Model 6). Each oral hygiene variable included in each model was maintained regardless of the final level of significance. Next, associations between self-reported toothbrushing frequency and the clinical oral hygiene indices were assessed using the chi-square test. All statistical analyses were performed with the aid of the SPSS Statistics™ program (SPSS for Windows, version 20.0, SPSS Inc., Chicago, IL, USA).

### Ethical considerations

This study received approval from the Araucaria Municipal Department of Education and Health and the Human Research Ethics Committee of the Federal University of Parana (process number 740.075.09.06). The parents/caregivers of the students signed a statement of informed consent agreeing to the participation of their children.

## Results

The final sample consisted of 589 students. The loss rate was 8.5%. In the calibration exercise, inter-examiner and intra-examiner Kappa coefficients were greater than 0.81 (very good) for all clinical measures, which is satisfactory for this type of evaluation [15].

Table 1 displays the descriptive analysis of DMFT index scores according to the independent variables. Adolescents with higher DMFT index were those with low household income and sugary foods/beverages consumption greater than three times per day. Furthermore, these adolescents presented poor OHI-S scores and high counts of mutans streptococci and lactobacilli.

**Table 1:** **Unadjusted assessment of oral hygiene variables association with prevalence of dental caries**

**Variables**	**DMFT**				
	**Mean**	**Std. Deviation**	**Median**	**Range**	***p*** **value**
**Gender**					
Male	2.24	2.15	2.00	0-10	0.289*
Female	2.46	2.41	2.00	0-15	
**Mother’s level of education**					
Up to 8 yrs	2.46	2.33	2.00	0-15	0.214*
More than 8 yrs	2.30	2.30	2.00	0-12	
**Household Income**					
≤2 BMW	2.83	2.58	2.00	0-15	0.002**
2< income ≤ 3 BMW	1.99	1.98	2.00	0-8	
> 3BMW	2.06	2.70	2.00	0-12	
**Frequency of toothbrushing**					
≤ twice a day	3.29	2.37	3.00	0-15	<0.001*
More than twice a day	1.14	1.54	0.00	0-7	
**No. of sugary foods consumed daily**					
7 or more	2.75	2.42	2.00	0-15	0.005**
4-6	2.37	2.34	2.00	0-12	
0-3	1.96	2.08	1.00	0-9	
**Mutans streptococci**					
≥ 10^5^UFC/mL	2.61	2.39	2.00	0-15	<0.001*
< 10^5^UFC/mL	1.80	2.01	1.00	0-10	
**Lactobacilli**					
≥ 10^5^UFC/mL	3.51	2.10	3.00	0-9	<0.001*
< 10^5^UFC/mL	2.28	2.30	2.00	0-15	
**Simplified Oral Hygiene Index**					
Unsatisfactory	2.80	2.44	2.00	0-15	<0.001*
Satisfactory	1.97	2.11	1.00	0-9	
**Visible Plaque Index**					
Present	2.79	2.41	2.00	0-15	<0.001*
Absent	1.75	2.02	1.00	0-9	

*Significance evaluated by Mann–Whitney test. **Significance evaluated by Kruskal-Wallis test. Results significant at the 5% level marked in bold. BMW: Brazilian Minimum Wage (about US$ 290.47 during the period of data gathering).

Table 2 displays the results of the multiple models for the determination of associations between dental caries and the different measures of oral hygiene. When the hygiene variables were incorporated into the multiple analyses separately, adolescents with a toothbrushing frequency less than twice a day (Model 1), unsatisfactory OHI-S scores (Model 2) and visible plaque (Model 3) had higher DMFT index scores. When the OHI-S was incorporated together with toothbrushing frequency, the former was non-significant due to the collinearity between these variables (Model 4). The same occurred when the VPI was incorporated into the model together with toothbrushing frequency (Model 5). Moreover, when the three variables were analyzed together (Model 6), only toothbrushing frequency retained statistical significance.

**Table 2:** **Multiple Poisson regression of association among independent variable and dental caries (DMFT)**

**Oral hygiene variables**	**Model 1**		**Model 2**		**Model 3**		**Model 4**		**Model 5**		**Model 6**	
	**RR [CI]**	p*	**RR [CI]**	p*	**RR [CI]**	p*	**RR [CI]**	p*	**RR [CI]**	p*	**RR [CI]**	p*
**Toothbrush Frequency**												
≤ twice a day	**2.67**						**2.80**		**2.79**		**2.83**	
	**[2.19-3.26]**						**[2.26-3.45]**		**[2.23-3.48]**		**[2.26-3.54]**	
More than twice a day	**1**	**<0.001**					**1**	**<0.001**	**1**	**<0.001**	**1**	**<0.001**
**Simplified Oral Hygiene Index**												
Unsatisfactory			**1.31**				0.90				0.92	
			**[1.11-1.54]**				[0.77-1.05]				[0.77-1.08]	
Satisfactory			**1**	**<0.001**			1	0.190			1	0.301
**Visible Plaque Index**												
Present					**1.48**				0.921		0.96	
					**[1.23-1.76]**				[0.78-1.09]		[0.80-1.16]	
Absent					**1**	**<0.001**			1	0.347	1	0.686

*Significance evaluated by Multiple Poisson regression. RR: Rate ratio adjusted by gender, mother’s level of education, household income, sugar intake, mutans streptococci and lactobacilli. CI: 95% confidence interval. Results significant at the 5% level marked in bold.

The analysis of toothbrushing frequency and the clinical oral hygiene indices revealed that adolescents who brushed their teeth less than twice a day exhibited visible plaque and unsatisfactory OHI-S scores (Table 3).

**Table 3:** **Association between self-reported toothbrushing frequency and clinical indexes for oral hygiene**

**Variables**	**Toothbrushing frequency**				***p*** value*
	**≤ twice a day**		**More than twice a day**		
	**n**	**(%)**	**n**	**(%)**	
**Visible Plaque Index**					
Present	277	(76.9)	83	(23.1)	**<0.001**
Absent	63	(27.5)	166	(72.5)	
**Simplified Oral Hygiene Index**					
Unsatisfactory	224	(79.7)	57	(20.3)	**<0.001**
Satisfactory	116	(37.7)	192	(63.3)	

*Chi-square test. Results significant at the 5% level marked in bold.

## Discussion

The present study demonstrates that self-reported toothbrushing frequency is a feasible alternative for measuring oral hygiene in epidemiological studies on dental caries in adolescents. No previous studies have addressed the possibility of using self-reported toothbrushing frequency by adolescents as a proxy measure of oral hygiene in place of clinical indices.

Oliveira, Watt and Hamer [16] raised the possibility of using self-reported toothbrushing frequency as a proxy measure of periodontal disease, which was associated with the risk of developing cardiovascular disease in a sample of adults in Scotland. However, no similar type of study was found regarding dental caries. Another study demonstrated a good level of agreement between the self-reports of children aged six to nine years and caregivers’ reports regarding oral health conditions, such as toothbrushing frequency, dental pain and dental treatment. The authors concluded that the use dental self-report measures for children appeared to be clinically valid and may be valuable in epidemiological investigations [17].

It is important to evaluate costs and benefits in the development of epidemiological studies. David et al. [11] found that information on self-reported oral health from 12-year-old adolescents was significantly associated with clinical conditions and could be used to assist in the planning of oral health programs in locations with limited resources.

The OHI-S index was first proposed in the 1960s [9] and is recommended by the World Health Organization to measure oral hygiene in epidemiological studies [15]. However, despite its simple proposal, the administration of this assessment tool requires a detailed clinical examination, time and a trained examiner, when evaluating a large number of individuals in an epidemiological study. The VPI is simplified alternative, as it does not classify the amount of plaque, but rather its presence or absence [10].

In the present study, when the oral hygiene measures were incorporated separately in the multiple models, significant associations were found with dental caries. Moreover, rate ratio values for the clinical indices were similar (Models 2 and 3), whereas the rate ratio value for Model 1 was nearly double the values found with Models 2 and 3, demonstrating that self-reported toothbrushing frequency is a good predictor, with a better capacity for discriminating between adolescents with different caries severity in comparison to the clinical indicators. Moreover, when all oral hygiene measures were incorporated together in the same model, only toothbrushing frequency was significantly associated with dental caries due to the collinearity of the different measures (Models 4 and 5). This is the most important result of the present study, as it demonstrates the viability of using self-reported toothbrushing frequency as a proxy measure of oral hygiene in epidemiological studies involving adolescents.

Independent of the evaluation method, adolescents with worse oral hygiene had higher DMFT index scores. This finding confirms data reported in the literature showing that oral hygiene habits are associated with dental caries experience in adolescents [2,5]. Poor oral hygiene leads to the accumulation of bacterial plaque. Acidogenic bacteria present in the early stage only contribute to mild, infrequent de-mineralization. However, when the presence of fermentable carbohydrates is frequent, a gradual increase in such bacteria occurs in the oral environment, causing an imbalance in the de-mineralization/mineralization process in dental tissues [18].

The present study has a limitation regarding the impossibility of assuming that self-reported toothbrushing frequency is a good proxy measure of oral hygiene in other age groups. Finally, it is important to consider that self-reported measures may be subject to respondent bias [19].

Despite the inherent error of self-reported measures, particularly when the toothbrushing frequency is assessed, it is important to bear in mind that if this error were constant over time, it could still be a good comparison parameter among epidemiological studies. An important aspect of the present study that may had minimized this limitation is that toothbrushing frequency was evaluated by a question addressing the times at which the adolescents brushed their teeth rather than a direct question addressing toothbrushing frequency. The aim of this strategy was to avoid the bias of a standard response that met the traditional orientation of brushing one’s teeth three times a day, and it may have contributed to an increase in the reliability of the data.

In the view of the findings and above considerations, one should use a clinical indicator when oral hygiene is the main explanatory variable, that is, when the aim of the study is to assess the association between oral hygiene and dental caries. In such cases, the Visible Plaque Index is suggested. However, if oral hygiene is only used for the adjustment of other explanatory variables for dental caries, self-reports from adolescents can be used, especially in large population-based surveys.

## Conclusion

Based on the present findings, self-reported toothbrushing frequency can be used as a proxy measure for clinical oral hygiene indices, which facilitates data collection in epidemiological studies addressing dental caries in adolescents.

## Abbreviations

DMFT: Decayed, missing and filled teeth index
OHI-S: Simplified oral health index
VPI: Visible plaque index
